# Development and internal validation of a nomogram for predicting postoperative bone nonunion in infectious spondylitis: a retrospective cohort study

**DOI:** 10.1080/07853890.2026.2709091

**Published:** 2026-07-28

**Authors:** Wentao Zhao, Lu Xing, Yongrui Yang, Wenkai Ruan, Jianlong Li, Rongpan Dang, Huigang An, Liang Xu, Yingxin Zhao, Hongdong Tan, Chenggui Zhang

**Affiliations:** Department of Orthopedics, Public Health Clinical Center Affiliated to Shandong University, Jinan City, Shandong Province, China

**Keywords:** Infectious spondylitis, bone nonunion, nomogram, predictive model, spinal fusion

## Abstract

**Background:**

Postoperative bone nonunion remains a major complication after instrumented fusion for infectious spondylitis, with substantially higher rates than in aseptic spinal surgery. Existingmodels lack validation and infection-specific predictors.

**Purpose:**

To develop and internally validate a nomogram for the perioperative prediction of postoperative bone nonunion ininfectious spondylitis patients.

**Method:**

This retrospective cohort study included 224 patients with infectious spondylitis undergoing posterior debridement and instrumented fusion (January 2020–December 2023). Patients were classified as union (*n* = 166) or nonunion (*n* = 58) by Bridwell grading on 12-month CT. After univariate screening, LASSO regression with the 1-SE criterion selected five predictors for multivariable logistic regression to construct the nomogram. Discrimination(AUC), calibration(Hosmer-Lemeshow test), and clinical utility(DCA) were assessed. Internal validation using 1,000 bootstrap resamples.

**Results:**

5predictors were incorporated: Lower Albumin (ALB) level, prolonged CRP normalization days, allogeneic bone graft materials, multilevel fusion and paravertebral abscess. The nomogram demonstrated excellent discrimination(AUC 0.936 ,95% CI: 0.896–0.975) and good calibration (*p* = 0.285). DCA indicated strongnet benefit across 2%-85% thresholds.Bootstrap validation (1,000 resamples) yieldedAUC0.94 (95% CI: 0.88–0.97) with excellent calibration (slope = 1.000, intercept ≈ 0.000, Emax = 0.061, Brier score = 0.077), confirmingminimal overfitting.

**Conclusion:**

A novel nomogram incorporating fiveperioperative predictors was developed and internally validated for early prediction of bone nonunion after fusion in infectious spondylitis, showing excellent discrimination, calibration, and clinical utility with minimal overfitting. External validation in larger multicenter cohorts is required beforeclinical application.

## Introduction

Infectious spondylitis, encompassing pyogenic, fungal, and tuberculous infections of the spine, represents a severe and debilitating condition that often requires surgical intervention when conservative management fails or neurological deficits occur [1,2]. Surgical treatment typically involves debridement, decompression, and instrumented spinal fusion to restore stability and eradicate infection [3,4]. Reported rates of postoperative bone nonunion in patients with infectious spondylitis range from 10% to 30%, which is substantially higher than the 5–15% typically observed in aseptic spinal fusion surgeries. Despite advances in surgical techniques and antimicrobial therapy, postoperative complications such as bone nonunion persist, leading to implant failure, recurrent deformity, and diminished functional recovery. The consequences of nonunion include persistent pain, spinal instability, hardware failure, progressive deformity, and increased risk of neurological deterioration, often necessitating complex revision surgeries. Management of nonunion in the setting of prior infection is particularly challenging due to compromised local biology, persistent inflammation, and the need for prolonged antimicrobial therapy and advanced bone grafting techniques.

Bone nonunion following spinal fusion surgery is influenced by a multitude of factors, including patient nutritional status, systemic inflammation, surgical approach, and graft materials. While several predictive models have been proposed for nonunion in aseptic spinal surgeries, their applicability to infectious spondylitis remains limited due to the distinct pathophysiology and clinical course of spinal infections. The lack of validated, infection-specific predictive tools represents a significant gap in clinical practice, hindering individualized risk assessment and preventive care. There are distinctions between the extremities and the spine, and the existing variables may not be directly applicable. Currently, there is a lack of predictive models for postoperative nonunion in spinal infections.

Nomograms have emerged as practical and intuitive tools for visualizing complex statistical models, enabling clinicians to estimate individualized probabilities of clinical outcomes based on multiple predictors [5–10]. Although nomograms have been developed for various spinal surgery outcomes, none have been specifically designed and validated for predicting bone nonunion in the context of infectious spondylitis.

Therefore, this study sought to develop and internally validate a multivariate nomogram for perioperative prediction of bone nonunion in patients undergoing instrumented fusion for infectious spondylitis. By integrating readily available clinical, laboratory, and imaging variables, this nomogram aims to provide a reliable, evidence-based tool for enhancing perioperative planning and improving postoperative outcomes in this challenging patient population.

## Materials and methods

### Patient selection and cohort formation

Consecutive patients with clinically or pathologically confirmed infectious spondylitis treated between January 2020 and December 2023 at our hospital. This research has been reported in line with the STROCSS criteria. This retrospective study was approved by the Institutional Review Board of Public Health Clinical Center Affiliated to Shandong University (Approval No. GWLCZXEC-SOP-K-2025-137). The requirement for informed consent was waived due to the retrospective nature of the analysis. Data analysis for this study began on 2026-02-15, after completion of data cleaning and database lock.

The inclusion criteria for this study were as follows: (1) Patients with infectious spondylitis confirmed by pathogen identification based on percutaneous biopsy of the lesion site. (2) Posterior surgical approach involving radical debridement and instrumented fusion with pedicle screw fixation. Patients treated with anterior-only, combined anterior-posterior, or minimally invasive approaches were systematically excluded to ensure cohort homogeneity. (3) Availability of complete perioperative clinical, laboratory, and radiological data. (4) Minimum follow-up of 12 months with CT scans to assess fusion status. Exclusion criteria were (1) To standardize the duration of preoperative antimicrobial therapy, patients with neurological deficits were excluded from this study. All patients received standardized pathogen directed preoperative therapy prior to surgery. (2) Patients with concurrent infections at other anatomical sites. (3) Active malignancy or metastatic spinal disease (4) Pathological fractures unrelated to infection. (5) Previous fusion surgery at the same spinal level. (6) Incomplete follow-up data.

**Figure 1.**
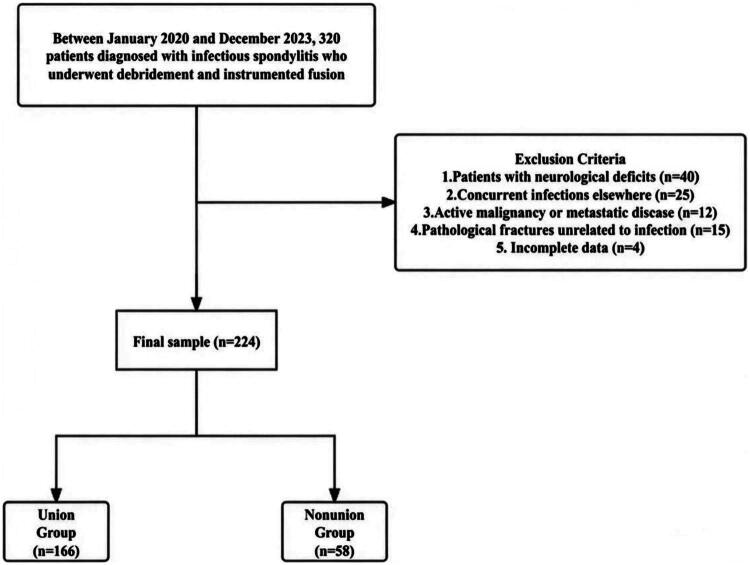
An illustration of this study flow chart.

A total of 320 patients were initially screened. After applying the inclusion and exclusion criteria, 96 patients were excluded (40 for neurological deficits, 25 for concurrent infections elsewhere, 12 for active malignancy or metastatic disease, 15 for pathological fractures unrelated to infection, and 4 were discharged against medical advice during hospitalization for personal reasons.) (Figure 1). This study finally included 224 patients, with union group (*n* = 166) and nonunion group (*n* = 58) according to Bridwell grade [11].

### Outcome definition

The primary outcome was postoperative bone nonunion, defined as the absence of continuous trabecular bone formation across the affected segment on follow-up imaging, with or without persistent mechanical instability, at the final follow-up. Fusion status was assessed at the final follow-up (minimum 12 months) using the Bridwell fusion grading criteria [11], where grades I-II indicate solid union and grades III-IV indicate nonunion.

### Data collection

Preoperative, intraoperative, and postoperative data were systematically extracted from electronic medical records (EMR) and Picture Archiving and Communication Systems (PACS) using a predefined data dictionary established prior to data collection. The data dictionary specified variable definitions, measurement units, and coding rules for categorical variables to ensure consistency and reproducibility across investigators. The collected variables included demographic characteristics, clinical factors, laboratory parameters, infection-related indicators, surgical variables, and radiological parameters.

Fusion status was assessed using postoperative CT scans obtained at the final follow-up. All CT images were independently evaluated by two experienced spine surgeons who were blinded to patients’ clinical characteristics, laboratory results, and predictor variables included in the model. Fusion was graded according to the Bridwell fusion grading system, with grades I–II classified as union and grades III–IV classified as nonunion.

Interobserver agreement for fusion grading was excellent (Cohen’s κ = 0.866). Discrepancies between reviewers were resolved through consensus discussion. Most disagreements occurred at the boundary between Bridwell grade II and grade III, which determines classification as union versus nonunion. When consensus could not be achieved initially, a senior spine surgeon performed adjudication according to predefined criteria.

In cases of discrepancy between the two investigators, the records were re-evaluated through consensus discussion. If disagreement persisted, adjudication was performed by a senior spine surgeon according to predefined criteria (*n* = 9, mainly Grade II vs. III). Patients with incomplete data were excluded according to the predefined inclusion and exclusion criteria, therefore, no missing data were present in the final statistical analysis.

### Perioperative management

All patients received standardized pathogen-directed antimicrobial therapy according to microbiological culture results and antimicrobial susceptibility testing. Patients with pyogenic infections received targeted intravenous antibiotics followed by oral therapy when appropriate, whereas patients with spinal tuberculosis received standard anti-tuberculosis chemotherapy. Surgical intervention was performed after adequate infection control and optimization of the patient’s general condition whenever clinically feasible.

All procedures were performed through a posterior-only approach. After radical debridement of infected and necrotic tissues, decompression was performed when necessary, followed by interbody bone grafting and pedicle screw fixation to restore spinal stability. Fusion levels were determined according to the extent of vertebral destruction, spinal instability, and involvement of adjacent segments. Multilevel fusion was defined as fusion involving more than three spinal segments.

The choice of graft material (autologous or allogeneic bone) was made according to the availability of autologous bone and local bone quality. Postoperatively, all patients received external brace protection for approximately 8–12 weeks and participated in a standardized rehabilitation program with gradual ambulation as tolerated.

Clinical and radiological follow-up evaluations were routinely performed at 3, 6, and 12 months after surgery and annually thereafter. Radiographs were obtained at each follow-up visit, while computed tomography (CT) was performed at a minimum of 12 months postoperatively or when fusion status was uncertain. Bone fusion was assessed according to the Bridwell fusion grading system.

### Statistical analysis

Continuous variables were presented as mean ± standard deviation or median (interquartile range), as appropriate, and categorical variables as frequencies and percentages. Comparisons between groups were performed using the Student’s t-test or Mann–Whitney U test for continuous variables and the chi-square or Fisher’s exact test for categorical variables.

Due to the limited sample size and imbalanced outcomes, the entire cohort was used for model development, with no separate training or validation split [12]. The final prediction model included two continuous predictors and three binary predictors, corresponding to five effective regression parameters. This yielded an events-per-parameter (EPP) ratio of approximately 11.6, exceeding the commonly recommended minimum threshold of 10 for logistic regression modeling. To mitigate overfitting, the least absolute shrinkage and selection operator (LASSO) regression was applied for variable selection, with the 1-standard error (1-SE) rule for lambda selection [13,14]. The selected predictors were subsequently entered into a multivariable logistic regression model for nomogram construction.

The predictive effect of the model was evaluated by calculating the area under the ROC curve (AUC). The calibration curve of the model was plotted using the Hosmer- Lemeshow goodness-of-fit test, with patients grouped into deciles according to predicted probabilities, and calibration plots comparing predicted and observed outcomes.

Internal validation of the model was performed using bootstrap resampling with 1,000 repetitions to estimate optimism-corrected performance metrics and assess model stability [15,16]. Decision curve analysis (DCA)was performed to estimate the clinical usefulness of the prediction model by quantifying the net benefits at different threshold probabilities.

Statistical analyses and image plotting were performed using R software (version 4.2.2; R Foundation for Statistical Computing, Vienna, Austria), *p* < 0.05, was considered statistically significant.

This study was reported in accordance with the Transparent Reporting of a multivariable prediction model for Individual Prognosis Or Diagnosis (TRIPOD) statement.

## Results

### Baseline characteristics

A total of 320 patients diagnosed with infectious spondylitis who underwent debridement and instrumented fusion between January 2020 and December 2023 were retrospectively reviewed. After applying the inclusion and exclusion criteria, 224 patients were included in the final analysis. Based on the Bridwell fusion grading system, patients were categorized into the union group (*n* = 166) and the nonunion group (*n* = 58). According to the Bridwell fusion grading system, the distribution of fusion grades among the 224 patients was as follows: Grade I in 98 (43.8%), Grade II in 68 (30.4%), Grade III in 42 (18.8%), and Grade IV in 16 (7.1%). Consequently, 166 patients (74.1%) were classified as having bone union (Bridwell Grades I–II) and 58 patients (25.9%) as having nonunion (Bridwell Grades III–IV). All patients had a median last follow-up time of 18 months (IQR: 15–22 months).

Demographic and clinical characteristics are summarized in Table 1. The union group comprised 61 (36.7%) females and 105 (63.3%) males, while the nonunion group included 25 (43.1%) females and 33 (56.9%) males. The mean age of the entire cohort was 60.8 ± 15.6 years, with the union and nonunion groups having mean ages of 58.6 ± 16.2 and 66.9 ± 11.8 years, respectively.

**Table 1.** Baseline demographic and clinical characteristics of patients with infectious spondylitis stratified by postoperative bone union status.

Variables	Total (*n* = 224)	Union Group (*n* = 166)	Nonunion Group (*n* = 58)	*p*	Entered Lasso
Pathogen, n (%)				0.053	No
Brucella	28 (12.5)	20 (12)	8 (13.8)		
Tuberculous	61 (27.2)	30 (18.1)	31 (53.4)		
Pyogenic	132 (58.9)	114 (68.7)	18 (31)		
Fungi	3 (1.3)	2 (1.2)	1 (1.7)		
Age, Mean ± SD	60.8 ± 15.6	58.6 ± 16.2	66.9 ± 11.8	0.001	Yes
Gender, n (%)				0.392	No
Female	86 (38.4)	61 (36.7)	25 (43.1)		
Male	138 (61.6)	105 (63.3)	33 (56.9)		
CRP Normalization Days, Mean ± SD	10.8 ± 5.0	9.4 ± 3.8	14.7 ± 6.1	< 0.001	Yes
Paravertebral Abscess, n (%)				< 0.001	Yes
No	173 (77.2)	141 (84.9)	32 (55.2)		
Yes	51 (22.8)	25 (15.1)	26 (44.8)		
Poor Healing, n (%)				0.699	No
No	200 (89.3)	149 (89.8)	51 (87.9)		
Yes	24 (10.7)	17 (10.2)	7 (12.1)		
Multilevel Fusion, n (%)				< 0.001	Yes
No	191 (85.3)	155 (93.4)	36 (62.1)		
Yes	33 (14.7)	11 (6.6)	22 (37.9)		
Bone Graft Materials, n (%)				< 0.001	Yes
Autologous Bone	145 (64.7)	124 (74.7)	21 (36.2)		
Allogeneic Bone	79 (35.3)	42 (25.3)	37 (63.8)		
Hypertension, n (%)				0.295	No
No	184 (82.1)	139 (83.7)	45 (77.6)		
Yes	40 (17.9)	27 (16.3)	13 (22.4)		
Diabetes, n (%)				0.101	No
No	179 (79.9)	137 (82.5)	42 (72.4)		
Yes	45 (20.1)	29 (17.5)	16 (27.6)		
Jumping Lesions, n (%)				0.021	Yes
No	188 (83.9)	145 (87.3)	43 (74.1)		
Yes	36 (16.1)	21 (12.7)	15 (25.9)		
WBC, Mean ± SD	7.1 ± 3.3	7.3 ± 3.5	6.5 ± 2.5	0.089	No
HB, Mean ± SD	114.8 ± 19.6	114.2 ± 18.8	116.7 ± 21.8	0.393	No
CRP, Median (IQR)	33.1 (10.2, 64.2)	38.4 (12.1, 68.3)	16.2 (8.0, 48.4)	0.035	No
ESR, Mean ± SD	53.2 ± 33.0	52.9 ± 32.5	54.2 ± 34.8	0.795	No
ALT, Median (IQR)	18.0 (12.0, 32.0)	19.0 (13.2, 33.0)	13.0 (11.0, 22.0)	0.135	No
AST, Median (IQR)	18.0 (13.0, 25.0)	19.0 (14.0, 27.5)	17.0 (13.0, 24.0)	0.312	No
ALB, Mean ± SD	36.6 ± 5.2	37.8 ± 4.6	33.1 ± 5.1	< 0.001	Yes
BUN, Median (IQR)	4.4 (3.5, 5.9)	4.5 (3.5, 5.9)	4.2 (3.2, 5.2)	0.153	No
CR, Median (IQR)	51.0 (43.0, 59.0)	51.0 (44.0, 59.0)	50.5 (43.0, 60.8)	0.918	No
Glu, Mean ± SD	5.6 ± 2.0	5.6 ± 2.1	5.8 ± 1.7	0.441	No
TG, Mean ± SD	1.4 ± 0.7	1.3 ± 0.7	1.4 ± 0.6	0.279	No
Chol, Mean ± SD	4.4 ± 1.1	4.3 ± 1.1	4.7 ± 1.0	0.037	Yes
HDL, Mean ± SD	1.1 ± 0.4	1.1 ± 0.4	1.2 ± 0.3	0.541	No
LDL, Mean ± SD	2.6 ± 0.9	2.6 ± 0.9	2.7 ± 0.9	0.19	No

CRP, C-reactive protein; ESR, erythrocyte sedimentation rate; WBC, white blood cell count; HB, hemoglobin; ALT, alanine aminotransferase; AST, aspartate aminotransferase; ALB, albumin; BUN, blood urea nitrogen; CR, creatinine; glu, glucose; TG, triglycerides; chol, total cholesterol; HDL, high-density lipoprotein; LDL, low-density lipoprotein; SD, standard deviation; IQR, interquartile range; or, odds ratio; CI, confidence interval. *Definitions:* CRP normalization days were defined as the number of days required for postoperative C-reactive protein levels to return to the normal range (<10mg/L). Poor healing was defined as impaired postoperative wound healing at the surgical site. Multilevel fusion was defined as fusion involving more than three spinal segments. Jumping lesions were defined as noncontiguous infectious lesions occurring at separate spinal levels.

### Clinical predictor selection

**Figure 2.**
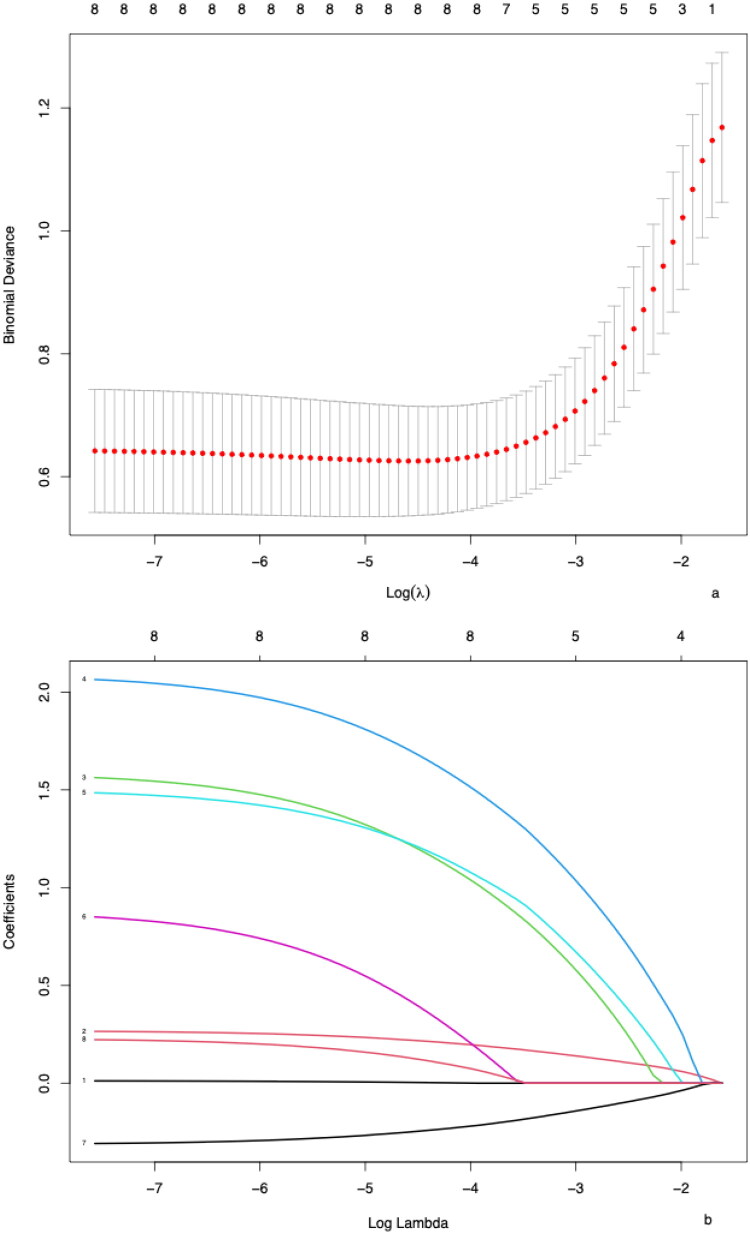
Screening variables by LASSO regression:(a) LASSO coefficient profiles of candidate predictors. Five non-zero coefficients were retained using the Lambda.1-SE criterion, which was selected to obtain a more parsimonious model and reduce overfitting. (b) Selection of the optimal penalty parameter (λ) using 10-fold cross-validated LASSO regression. The left dashed line indicates the value of λ that achieved the minimum cross-validation error (Lambda.min), whereas the right dashed line represents the largest λ within one standard error of the minimum error (Lambda.1-SE). The Lambda.1-SE criterion was used for variable selection in the final model.

A total of 25 perioperative clinical, laboratory, and radiological variables were initially considered. Univariate logistic regression identified 8 variables significantly associated with nonunion. To avoid overfitting and enhance model generalizability, these variables were subsequently subjected to LASSO regression (Figure 2). Using the 1-standard-error (1-SE) criterion, five non-redundant predictors were retained: ALB, CRP normalization days, Bone graft materials, Multilevel fusion, Paravertebral abscess (Table 2).

**Table 2.** Multivariate regression model based on LASSO regression results.

Variable	B	SE	OR	CI	*p*
Intercept (Constant)	5.443	1.803	–	–	<0.001
,	0.263	0.054	1.24	(1.15 ∼ 1.34)	<0.001
Paravertebral abscess (Yes vs. No)	1.513	0.526	4.58	(2.35 ∼ 8.95)	<0.001
Multilevel fusion (Yes vs. No)	2.065	0.613	8.61	(3.83 ∼ 19.35)	<0.001
Bone Graft Materials (Allogenic vs. Autologous)	1.6	0.487	5.2	(2.74 ∼ 9.86)	<0.001
ALB (per 1 g/L increase)	−0.316	0.061	0.82	(0.76 ∼ 0.88)	<0.001

Reference categories/units: CRP normalization days (per 1-day increase); Paravertebral abscess (yes vs. No); Multilevel fusion (yes vs. No); bone graft materials (allogeneic vs. autologous); albumin (per 1 g/L increase).

### Nomogram construction

**Figure 3.**
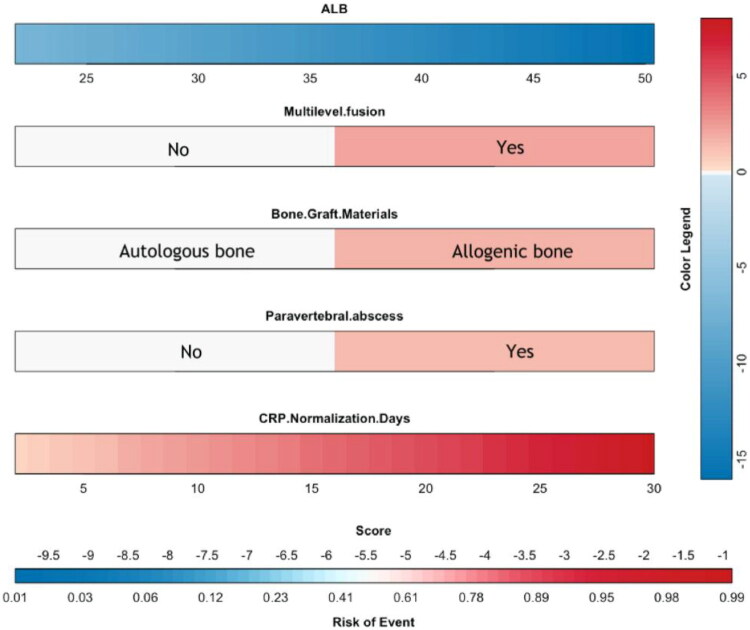
Nomogram for Postoperative Bone Nonunion in Patients with Infectious Spondylitis.

A multivariate logistic regression model was built using the five selected predictors. Based on this model, a nomogram was constructed to provide a visual tool for individualized risk estimation. Each predictor was assigned a score on a point scale, the sum of these scores corresponds to a total point value, which can be translated into a predicted probability of postoperative bone nonunion (Figure 3).

### Performance and validation of the nomogram

The nomogram demonstrated excellent discriminative ability, with an area under the ROC curve (AUC) of 0.936 (95% CI: 0.896–0.975). Calibration was assessed using the Hosmer–Lemeshow test (*p* = 0.285), and the calibration plot indicated strong agreement between predicted and observed outcomes, confirming model robustness.

Internal validation was performed using 1,000 bootstrap resamples. The optimism-corrected AUC (apparent C-index) was 0.940 (95% CI: 0.880–0.970), indicating minimal overfitting. Calibration was excellent, with a calibration slope of 1.000 and intercept of ≈ 0.000. The maximum absolute calibration error (Emax) was 0.061, and the Brier score was 0.077, reflecting good overall predictive accuracy. The calibration plot (Figure 4b), generated with loess smoothing and bootstrap bias correction, showed close agreement between predicted and observed probabilities of postoperative bone nonunion across the range of risk.

Figure 4.(a) Receiver Operating Characteristic (ROC) Curve of the Nomogram. The 95% confidence interval for the area under the curve (AUC) is provided in parentheses. (b) Calibration Curve of the Nomogram. The apparent curve illustrates the association between predicted and observed probabilities of post-op bone nonunion, with the bias-corrected curve derived from 1000 bootstrap resamples. The ideal curve, a 45° line, represents perfect predictive accuracy. (c) Decision Curve Analysis (DCA) of the Nomogram. The red solid line represents the net benefit of using the nomogram to guide clinical decisions. The model demonstrates clinical utility (net benefit above both ‘treat all’ and ‘treat none’ reference lines) across a broad range of threshold probabilities (approximately 2% to 85%). The model demonstrates clinical utility (net benefit above both ‘treat all’ and ‘treat none’ reference lines) across this wide range. However, the decision to implement specific interventions at any given threshold must be individualized through clinical judgment, taking into account the full clinical context and patient-specific factors.

**Figure:**
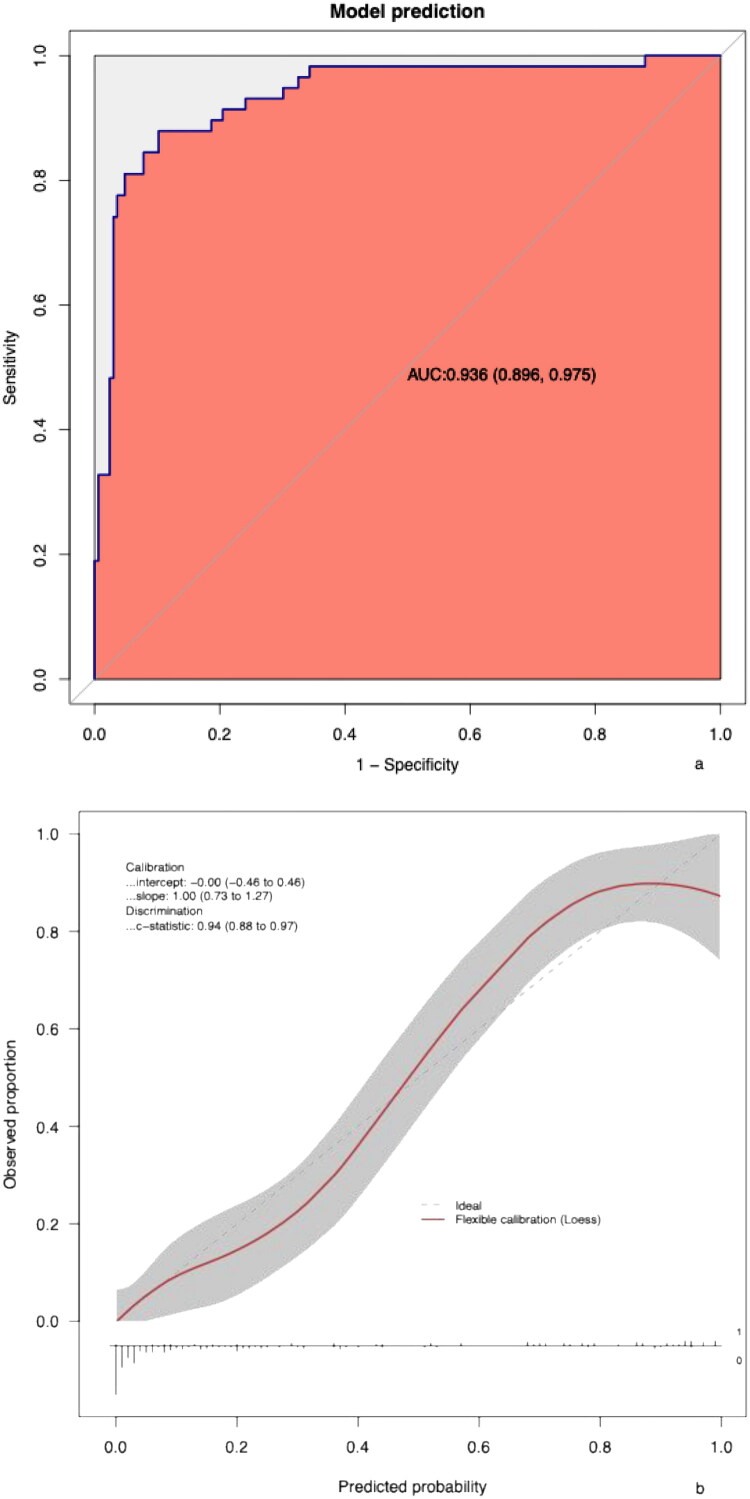

**Figure:**
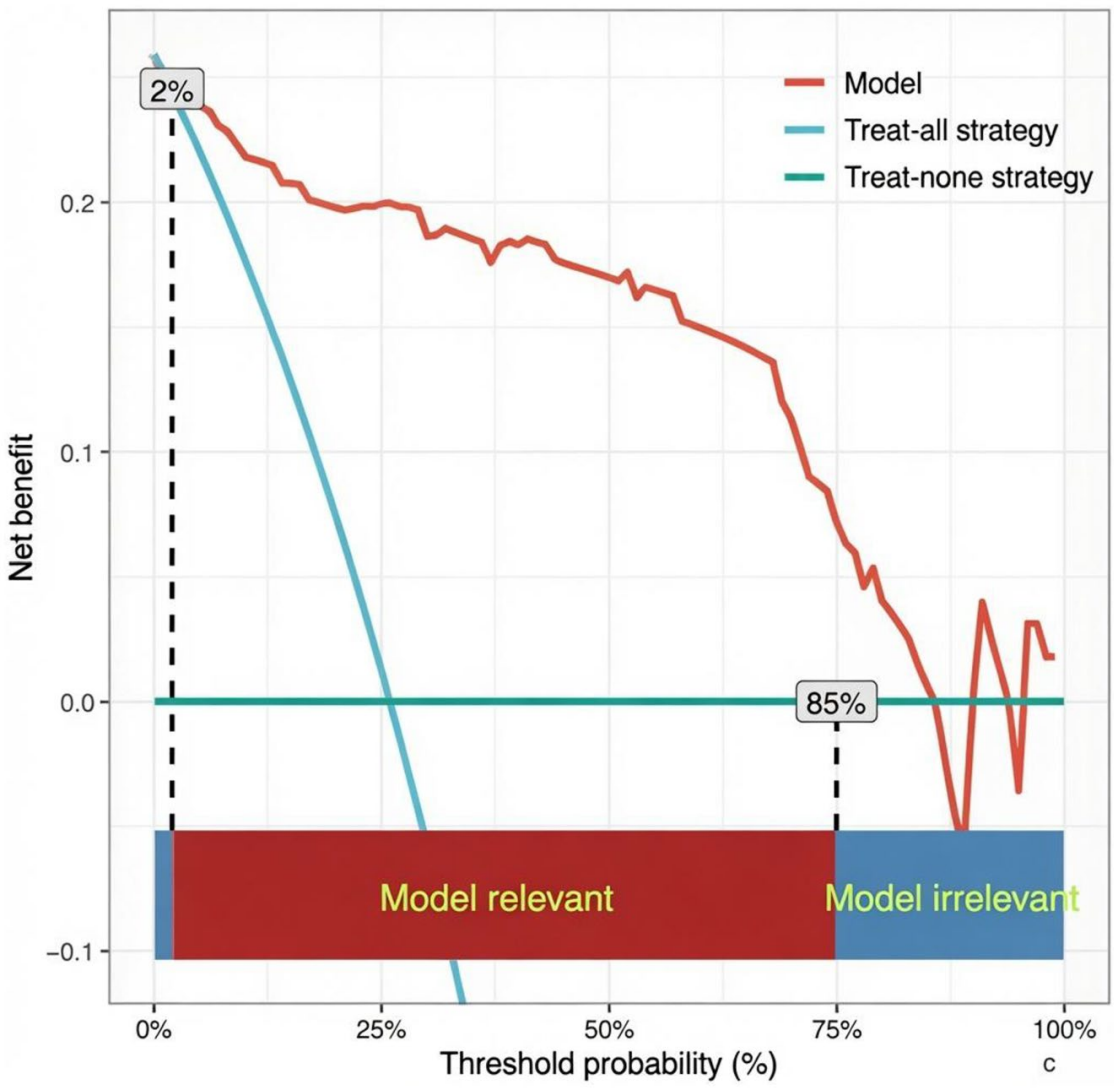

Decision curve analysis (DCA) revealed that the nomogram provided significant net clinical benefit across a wide range of threshold probabilities, supporting its utility in clinical decision-making (Figure 4).

## Discussion

The present study developed and internally validated a practical nomogram integrating five independent perioperative predictors, lower serum albumin level, prolonged CRP normalization time, use of allogeneic bone graft material, multilevel spinal fusion, and presence of paravertebral abscess, for early prediction of postoperative bone nonunion after instrumented fusion in patients with infectious spondylitis. This tool achieved excellent discrimination (AUC 0.936) and calibration, with internal bootstrap validation (1,000 resamples) demonstrating minimal optimism., indicating limited evidence of overfitting and good overall performance despite the modest sample size. These results address a longstanding unmet need, as prior predictive models for spinal nonunion were derived almost exclusively from aseptic degenerative or deformity cohorts and fail to capture the unique pathophysiological milieu of persistent inflammation, malnutrition, and impaired osteogenesis that characterizes infectious spondylitis.

Each predictor identified in the multivariable model has strong biological plausibility and aligns with mechanistic evidence from the spinal infection literature. Hypoalbuminemia serves as a surrogate for malnutrition and systemic catabolism, both of which compromise osteoblast function, collagen synthesis, and callus formation. Consistent with this, preoperative hypoalbuminemia has been independently linked to increased risks of deep infection, revision surgery, and poor healing after lumbar fusion procedures [17].

Prolonged time to CRP normalization, in turn, reflects incomplete resolution of the inflammatory cascade and ongoing bacterial burden, which delays the transition from the inflammatory to the reparative phase of bone healing. Studies monitoring serial CRP kinetics in pyogenic spondylitis and instrumented spinal surgery have similarly shown that delayed normalization or secondary elevations strongly predict persistent infection and subsequent complications [18–21].

The use of allogeneic bone graft emerged as a potent risk factor (OR 5.2), likely because allografts depend entirely on host osteoinduction and revascularization, processes already compromised by the infected, avascular, and inflamed local environment. Comparative studies in instrumented anterior cervical fusion have documented significantly slower radiographic union with allografts versus autografts, although long-term fusion rates may converge in non-infected settings, in the presence of infection, this temporal disadvantage appears amplified [22,23].

Multilevel fusion (OR 8.61) increases both the surface area requiring biological incorporation and the biomechanical demands on the construct, thereby elevating shear forces and micromotion at graft–host interfaces. This finding echoes reports in pyogenic spondylitis cohorts, where longer instrumentation constructs and multilevel involvement were associated with higher rates of revision for pseudarthrosis [23–25].

Finally, paravertebral abscess signifies extensive local tissue destruction and devascularization, which impairs nutrient delivery and graft incorporation; extensive abscess formation has been repeatedly associated with more aggressive disease courses and higher complication rates in both pyogenic and tuberculous spondylitis.

Strengths of this work include the use of a standardized, objective outcome, comprehensive candidate variable screening, and state-of-the-art statistical validation techniques that account for the relatively small nonunion subgroup. The DCA results further support the translational value of our nomogram. By identifying patients with predicted nonunion risk in the actionable window, clinicians can tailor perioperative strategies to individual risk profiles, such as preferential autologous bone grafting, nutritional prehabilitation, extended antimicrobial therapy, and intensified surveillance, potentially reducing the incidence of this challenging complication and associated revision surgeries. This aligns with the growing emphasis on personalized medicine in spinal infection management. Nevertheless, several limitations warrant acknowledgment. It relied on complete-case analysis of patients with pathogen confirmation by percutaneous biopsy, which may introduce selection bias and limit generalizability to culture-negative or clinically diagnosed cases. Patients with neurological deficits were excluded to standardize preoperative antimicrobial therapy, potentially creating spectrum bias by omitting more severe disease presentations. The choice of bone graft material was determined by clinical judgment rather than randomization, raising the possibility of treatment-selection bias. Although internal bootstrap validation demonstrated minimal optimism, external validation was not performed. Several potentially important predictors of bone healing, including smoking status, bone mineral density, osteoporosis, detailed glycemic control, organism-specific factors, and granular surgical parameters, were unavailable. Furthermore, despite good inter-rater reliability for fusion assessment, some misclassification risk remains at the boundary between Bridwell grades II and III. These limitations highlight the need for prospective multicenter validation with more comprehensive data collection before wider clinical application.

Finally, the model does not yet incorporate emerging biomarkers or advanced imaging metrics that could further refine predictive accuracy.

## Risk of bias and applicability assessment

We evaluated the risk of bias and applicability of our prediction model study using the PROBAST framework across four domains. Participants domain: Low risk of bias. We included consecutive patients with infectious spondylitis who underwent posterior debridement and instrumented fusion during a defined study period, with clear inclusion and exclusion criteria applied systematically. However, the single-center design and exclusion of patients with neurological deficits (to ensure standardized preoperative antimicrobial duration) may have restricted the spectrum of disease severity, introducing a moderate concern regarding applicability to the broader population of infectious spondylitis patients. Predictors domain: Low risk of bias. Candidate predictors were selected a priori on the basis of clinical relevance and literature, and all variables (demographic, laboratory, and imaging) were objectively defined and routinely available in the perioperative period. Outcome domain: Low risk of bias. The primary outcome (postoperative bone nonunion) was assessed at a minimum of 12 months using standardized Bridwell fusion grading criteria on CT scans. Two experienced spine surgeons blinded to clinical and laboratory data performed the grading independently, with excellent inter-rater reliability (Cohen’s κ = 0.866). Analysis domain: Moderate risk of bias. The model was developed and internally validated on the full cohort using LASSO regression with the 1-SE criterion for variable selection, followed by multivariable logistic regression and rigorous internal validation with 1000 bootstrap resamples. The events-per-predictor ratio was adequate (approximately 11.6). Optimism-corrected performance metrics indicated minimal overfitting. Nevertheless, the retrospective single-center design, modest event rate (*n* = 58), and absence of external validation increase the potential for overfitting and limit transportability to other centers. We mitigated these risks through state-of-the-art statistical methods (LASSO + bootstrap) and decision curve analysis. External validation in larger, multicenter prospective cohorts remains essential before widespread clinical application. Overall, the study has low risk of bias in the participants, predictors, and outcome domains, and moderate risk of bias in the analysis domain, primarily due to the lack of external validation. The model demonstrates good internal validity and clinical utility within the studied population but should be applied cautiously until further validated.

In summary, the developed nomogram represents a novel, internally validated tool for perioperative risk stratification in patients with infectious spondylitis. While the model shows promising discriminatory performance, its clinical utility and potential impact on patient outcomes require prospective validation and dedicate

## Conclusion

In conclusion, this study developed and internally validated a novel nomogram for predicting postoperative bone nonunion after instrumented fusion in patients with infectious spondylitis. By incorporating infection-specific risk factors, this practical tool may facilitate early identification of patients at elevated predicted risk of nonunion. Whether risk-stratified perioperative interventions can reduce nonunion-related morbidity remains a hypothesis that requires prospective validation and clinical-impact studies before any definitive conclusions can be drawn. Limitations of the retrospective single-center design and modest sample size necessitate multicenter prospective validation before routine clinical use.

## Data Availability

The datasets used and/or analyzed during the current study are available from the corresponding author on reasonable request.
